# Statistical analysis plan for the Dual mTorc Inhibition in advanCed/recurrent Epithelial ovarian, fallopian tube or primary peritoneal cancer (of clear cell, endometrioid and high-grade serous type, and carcinosarcoma) trial (DICE)

**DOI:** 10.1186/s13063-021-05669-9

**Published:** 2022-01-05

**Authors:** Consuelo Nóhpal de la Rosa, Jonathan Krell, Emily Day, Aaron Clarke, Meena Reddi, Lee Webber, Francesca Fiorentino

**Affiliations:** 1 Imperial Clinical Trials Unit, School of Public Health, Imperial College London, London, UK; 2 Department of Surgery and Cancer, Imperial College London, London, UK; 3 Nightingale-Saunders Clinical Trials & Epidemiology Unit, King’s CTU, King’s College London, London, UK

**Keywords:** Statistical analysis plan, Randomised controlled trial, Ovarian cancer, Platinum-resistant, TAK228, Paclitaxel

## Abstract

**Background:**

Treatment for ovarian cancer includes platinum-based chemotherapy, but many women become resistant to chemotherapy, becoming platinum-resistant. Standard of care for these women is weekly paclitaxel chemotherapy, but cancers can often become paclitaxel resistant. TAK228, an investigational dual TORC1/2 inhibitor, is an oral therapy that can be added to standard treatment. The DICE trial is a phase II international multicentre, parallel-group, superiority clinical trial with 1:1, open label randomisation which has the aim of investigating the effectiveness of TAK228 plus weekly paclitaxel. The planned sample size is 124 women (62 per treatment arm) with platinum-resistant ovarian cancer.

**Objective:**

To outline the planned analyses for DICE in a statistical analysis plan (SAP) before database hard lock and the start of analysis. This ensures that bias is minimised during the analysis phase.

**Results:**

This SAP provides detailed descriptions of the analysis principles and statistical procedures for analysing primary and secondary outcomes of the trial. The primary outcome is overall progression-free survival (PFS). Secondary outcomes include progression-free survival (PFS) at 24 weeks, overall response rate (ORR), duration of response (DoR), time to progression (TTP), clinical benefit rate (CBR) at 4 months, Cancer Antigen 125 (CA125) response according to Gynaecological Cancer Intergroup (GCIG) criteria, overall survival (OS), safety and tolerability as assessed by adverse events and the quality-of-life questionnaires (EORTC QLQ-C30 and EORTC QLQ-OV28). This detailed description includes significance levels, sensitivity analyses and compliance analysis.

**Discussion:**

The DICE trial will determine whether the addition of TAK228 to weekly paclitaxel chemotherapy shows a statistically significant improvement to participant’s progression free and overall survival and that the adverse events (AEs) and quality of life (QoL) are not significantly worse than the standard treatment.

The study commenced recruitment in September 2018. An interim analysis was performed in early 2021, the results of which advised continuation of the trial. The study recruitment is ongoing and is due to complete by the end of 2021.

**Trial registration:**

ClinicalTrials.govNCT03648489. Registered on 27 August 2018

**Supplementary Information:**

The online version contains supplementary material available at 10.1186/s13063-021-05669-9.

## Introduction

The Dual mTorc Inhibition in advanced/recurrent epithelial ovarian, fallopian tube or primary peritoneal cancer (of clear cell, endometrioid and high-grade serous type, and carcinosarcoma) trial (DICE) is a randomised control trial that investigates the effects of TAK228 plus weekly paclitaxel for women with ovarian cancer. This trial is a phase II international multicentre, parallel-group, superiority clinical trial with 1:1, open label randomisation conducted in thirteen centres in the UK and Germany. A protocol paper (version 7) describing the design of DICE has previously been published [1].

This paper describes the statistical analysis plan (SAP) for the DICE trial. In accordance with good clinical practice (GCP) [2], the SAP was developed, completed and approved before the final database hard lock and the initiation of the final data analysis. The first patient was randomised on 21 September 2018. The study will end 6 months after the last patient stops treatment due to disease progression, unacceptable toxicity or withdrawal. The chair and the independent statistician from the Independent Data Monitoring Committee (IDMC) have approved this SAP (Statistical Analysis Plan version 1, 2 June 2021).

### The clinical study background and rationale

Standard treatment for ovarian cancer is surgery to remove the womb, ovaries and tubes, followed by chemotherapy using the platinum-based drug carboplatin and a taxane called paclitaxel. A small proportion of women are cured; however, in 80–90% of the cases, the cancer grows or comes back in recurrent/relapsed ovarian cancer. Of these recurrent ovarian cancer cases, 20% are known as platinum-resistant, meaning that they relapse either during chemotherapy or within 6 months after chemotherapy. Platinum-resistant women are the population under study in the DICE trial.

There is a lack of effective treatments for this population. As platinum-resistant cancer can also become resistant to paclitaxel, there is an unmet need to improve the effectiveness of chemotherapy by combining it with additional treatment.

One promising additional treatment is TAK228 which is an oral targeted therapy that blocks the phosphoinositide 3-kinase (PI3K)/protein kinase B (AKT)/mammalian target of rapamycin (mTOR) pathway by inhibiting the molecules mTORC1 and mTORC2. The combination of TAK228 and paclitaxel is considered investigational.

In summary, the DICE study investigates whether TAK228 and weekly paclitaxel is better than weekly paclitaxel alone to improve overall progression-free survival in women with ovarian cancer.

## Outcomes

### Primary outcome

The primary outcome evaluated in this trial is progression-free survival (PFS), which is defined as the time in months from randomisation to first evidence of disease progression or death due to any cause. PFS will be measured at the end of the study. This outcome is used to compare the efficacy of the combination of TAK228 plus paclitaxel to paclitaxel alone. PFS is evaluated by the Response Evaluation Criteria in Solid Tumours (RECIST, version 1.1) [3] which is based on radiological imaging using computerised tomography (CT) or magnetic resonance imaging (MRI) scans. Data is obtained at baseline, followed by assessment every 8 weeks during the assigned treatment after randomisation, at the end of treatment, and finally every 3 months during follow-up. Patients who do not have baseline disease assessment are left censored, whereas patients are right censored if:

- The patient is alive and does not have documentation of disease progression before the date of completion of the study.
- The patient withdraws from the trial during the study treatment period and asks to stop being followed up.
- A patient is lost to follow-up.

### Secondary outcomes

Secondary outcomes include PFS at 24 weeks and several others measures including the following: overall response rate (ORR), duration of response (DoR), time to progression (TTP), clinical benefit rate (CBR) at 4 months, response according to Gynaecologic Cancer Intergroup (GCIG) criteria, overall survival (OS), safety and tolerability and two quality of life questionnaires from the European Organisation for Research and Treatment of Cancer (EORTC), namely the QLQ-C30 [4] core questionnaire and the supplementary questionnaire QLQ-OV28 for ovarian cancer. All these outcomes are used to compare the efficacy of TAK228 plus paclitaxel to paclitaxel alone. As for the primary outcomes, the secondary time to event outcomes (PFS at 24 weeks, DoR, TTP), as well as other secondary outcomes (ORR and CBR at 4 months) are evaluated by the Response Evaluation Criteria in Tumours (RECIST, version 1.1) [3]. Each time to event outcome will be censored according to its definition. Additional file 1: Appendix 1 contains definitions of the time to events outcomes and censoring point for each outcome. Definitions of other secondary outcomes are also included in Additional file 1: Appendix 1. These are also listed in the protocol [1].

## Primary estimand

The addendum to the ICH E9 on estimands and sensitivity analysis in clinical trials [5] “presents a structured framework to formulate clinical trial objectives, design, conduct, analysis and interpretation, … the treatment effect(s) of interest that a clinical trial should address. Precision in describing a treatment effect of interest is facilitated by constructing the “estimand” corresponding to a clinical question of interest.”

In the DICE trial, the estimand aims to answer the specific research question: does TAK228 added to paclitaxel improve the progression-free survival of women with cancer of the fallopian tube, ovaries or peritoneum, resistant to platinum-based chemotherapy compared to paclitaxel alone?

### Primary estimand attributes

- Treatment: TAK228 added to paclitaxel compared to paclitaxel alone (control).
- Population: women with cancer of the fallopian tube, ovaries or peritoneum, that is resistant to platinum-based chemotherapy. Patients are selected using the inclusion/exclusion criteria listed in the Protocol [[1].
- Variable: progression-free survival defined as the time in months from randomisation to first evidence of disease progression or death due to any cause, as assessed at the end of study.
- Intercurrent events: We anticipate the following intercurrent events: treatment compliance and treatment modification. Treatment compliance is determined by patient behaviour whilst treatment modification is determined by the clinical and pharmacy team.
  - Treatment compliance.
    In the DICE trial, the patients are given two bottles of IMP every two cycles to cover their daily doses, with additional capsules contained in the two bottles. The bottles are returned to the pharmacy at the end of the two cycles and capsules are counted to measure compliance with the dosing regimen. Compliance is defined as a patient not having any modification to the assigned treatment as stipulated by the protocol. Compliance is estimated as a rate of capsule consumption across all days for each cycle (as a continuous variable). Further, each patient will be classified as a ‘complier’ based on whether their compliance rate is above or below a set threshold. Two thresholds will be used to test the effect of compliance on the primary outcome: 80% and 90%.
  - Treatment modification
    Treatment modification is defined as any incorrect dose, omitted dose or dose reduction as decided by the clinical team. Hence, ‘unmodified treatment’ (cycle days without treatment modification) will be estimated as a rate (unmodified treatment rate) across all days (as a continuous variable), and patients will be classified as having ‘unmodified treatment’ based on whether their unmodified treatment rate is above or below a set threshold. The effect of treatment modification will be investigated using thresholds of 80% and 90%.
- Population level summary: difference in progression-free survival. The primary analysis to confirm this hypothesis will be between arms (Paclitaxel and TAK228) Kaplan-Meier curves and Cox model hazard ratio as a measure of the difference in progression-free survival adjusted by age and the randomisation stratification factors.

## Trial design

The DICE trial is an open-label, phase II international multi-centre randomised parallel group (1:1) clinical trial, with a superiority framework. It will be performed in 13 hospitals in the United Kingdom (UK) and in Germany.

### Recruitment and eligibility criteria

Recruitment takes place as part of hospital outpatient clinic visits at participating sites. To confirm eligibility, informed consent is obtained before the patient undergoes screening within the 28 days prior to randomisation (as per Additional file 4: Supplement 1—Table 1).

Eligible patients are included in the trial only if they meet all the inclusion criteria and none of the exclusion criteria as listed in the study protocol [1]. Once they are cleared for inclusion, they are randomised.

### Randomisation

Randomisation, based on a 1:1 ratio, is performed centrally via the InForm database, using stratified and blocked randomisation, with random block sizes. There are three stratification factors: non-serous (clear cell or endometrioid) vs serous (high grade serous or carcinosarcoma) cancer, number of prior lines of chemotherapy (≤ 2 vs > 2 lines) and prior taxane interval (< 6 months vs ≥ 6 months or no prior taxane).

### Treatment and assessment

The treatment regimen for the patients, who are allocated either paclitaxel alone or paclitaxel plus TAK228, must commence within 7 calendar days after randomisation. Patients will receive the treatment until disease progression, unacceptable toxicity or withdrawal is recorded.

The assessments performed during the study for paclitaxel alone or paclitaxel plus TAK228 are presented in detail in Table 2 and Table 3 of Additional file 4: Supplement 1. The study will be terminated 6 months after the last patient receiving study treatment stops that treatment.

### Withdrawal from study

Reasons for withdrawal, such as patient decision, loss to follow-up, death, AEs, investigator decision, pregnancy and/or breast feeding/lactation, will be reported. Also, if a patient dies, the cause of death will be reported.

## Sample size

The sample size calculation is based on the hypothesis that the administration of TAK228 in combination with paclitaxel will increase the median PFS by 2.15 months (from 4 to 6.15 months (hazard ratio of 0.65)) when compared to paclitaxel alone. The trial is designed to detect this difference over an 18-month accrual period (12 months recruitment and 6 months follow-up), with 80% power and a type I error rate of 10% (one-sided), no loss to follow-up and a minimum 6-month follow-up per participant.

Based on an initial sample size calculation using the SAS software and using the log-rank test, a total of 118 participants (59 per treatment arm) are needed to achieve 97 progression or death events. However, this does not consider the interim analysis for futility and safety. An interim analysis to assess safety and futility was planned when approximately 50% of the total progression events occurred. Hence, the final sample size was adjusted to account for the statistical analysis method used at interim analysis [6, 7]. The group sequential method of O’Brien Fleming (OF) [8] was used to select stopping rules for futility based on the primary endpoint analysis. Accordingly, in order to maintain power for the final analysis, allowing for one interim analysis of futility and safety, the number of events needed is 102 (see Additional file 2: Appendix 2 for more details). To attain this number of events, the adjusted sample size is 124 participants (62 per treatment arm).

Patients who withdraw or are withdrawn before their allocated intervention is administered will be replaced.

### Statistical interim analysis

The interim analysis was performed during the study, after 49 PFS events (approximately 50% of the expected events) occurred. Based on the results of the interim analysis, the IDMC advised to continue the study to completion. More information on the interim analysis can be found in Additional file 2: Appendix 2 (Interim Analysis).

## Analysis considerations for the final statistical analysis

### Patient flow diagram

The number of patients screened, consented and randomised for each treatment arm (paclitaxel alone and paclitaxel plus TAK228) will be presented according to the Consolidate Standards of Reporting Trial (CONSORT) [9] diagram (Figure 1 in Additional file 5: Supplement 2). This will also report the number of patients that progress to each stage of the treatment cycle as well as the number of patients that experience disease progression, unacceptable toxicity or withdrawal within each cycle.

### Analysis populations

All analyses will be performed on an intention-to-treat (ITT) basis to include all participants who were randomised. A modified intention-to-treat (mITT) analysis will be carried out which excludes patients who withdrew or are withdrawn before any intervention is administered. Patients in the ITT and mITT populations will be included in the treatment arm to which they were randomised. Analysis for the secondary outcomes concerned with safety and tolerability will be carried out on all patients receiving at least one dose of the allocated treatment (mITT population).

### Analysis principles

All summaries and analysis will be conducted using the ITT population unless otherwise specified. The statistical significance for the primary outcome is set at 11.005% (one-sided) to allow for an interim analysis of the primary endpoint (details in Additional file 2: Appendix 2).

For the comparison between the two treatment arms (paclitaxel alone or paclitaxel plus TAK228) for all secondary endpoints a two-tailed 5% significance level will be used. All effect estimates will be reported with a 2-sided 95% confidence interval.

The final analysis will be performed once the data is cleaned, and the database is hard locked. The statistical package STATA version 17 will be used to conduct all the analyses listed in the SAP.

Summaries of continuous variables will be presented as means and standard deviations if normally distributed and as medians and inter-quartile ranges for skewed data. Categorical variables will be presented as frequencies and percentages in the form of summary tables.

For each time to event outcome, the number and percentage of patients who had the event, were censored, withdrew or were lost to follow-up will be estimated.

### Derived variables

#### Treatment modification and compliance

As lack of compliance and adherence to cancer medications has been found to be associated with worse outcomes [10], treatment modification and compliance will be included in the analysis to address if compliance has an impact on the effectiveness of the assigned treatment (see the ‘Primary estimand’ section for a definition of compliance and treatment modification).

Patients will be classified as having ‘unmodified treatment’ based on whether their unmodified treatment rate is above or below two thresholds, 80% and 90%, and the effect of treatment modification investigated. Results for both thresholds will be reported.

Patient will be classified as a ‘compliers’ based on whether their compliance rate is above or below two thresholds, 80% and 90%, and the effect of compliance on the primary outcome will be tested. Results for both thresholds will be reported.

If a patient drops out, modification and compliance will only be reported until the date that they withdraw from the study.

#### COVID-19

Given that the DICE trial is recruiting during the COVID-19 pandemic, the impact of the pandemic on the subject population of the trial will be assessed [11]. On March 24, 2020, the first UK lockdown started, and so this date is used to define the start of the COVID-19 pandemic.

## Statistical analysis

The baseline characteristics, laboratory parameters, and outcome of physical examinations will be summarised for paclitaxel alone and paclitaxel plus TAK228 (as described above in the ‘Analysis principles’ section). The list of baseline characteristics and laboratory parameters that will be included in the final analysis are found in Additional file 4: Supplement 1—Tables 4 and 5 (respectively).

### Analysis of the primary outcome

The primary analysis of progression-free survival (PFS) will be a log-rank test adjusted by the stratification factors to estimate the difference, between groups, in time (in months) from randomisation to the earliest sign of disease progression or death due to any cause. Patients are right and left censored as described above in the ‘Primary outcome’ section. Kaplan-Meier curves and median PFS, and the corresponding 95% confidence intervals, will be estimated. The hazard ratio (HR), estimated using a Cox proportional hazards model adjusted by treatment arm, age, and stratification factors and its 95% CI will also be calculated to examine the effects of prognostic factors. Statistical significance for the primary outcome is set at 11.005%.

### Sensitivity analyses of the primary outcome

Five separate sensitivity analyses for the primary outcome will be conducted to test the robustness of the conclusions made from the primary analysis of the primary outcome.

A sensitivity analysis, using the primary outcome methods, will be completed based on excluding patients that have:

1. A dose omitted and/or dose reduced,
2. Not complied with treatment (complied to below the threshold of compliance),
3. Withdrawn from the study,
4. Protocol violation and/or deviation due to non-adherence to treatment,
5. Protocol violation and/or deviation due to COVID-19.

A non-stratified log-rank test will be used for the sensitivity analyses if the number of patients within the strata is too small [12].

### Analyses of secondary outcomes

For the time to event secondary outcomes (PFS at 24 weeks, DoR, TTP and OS), two-sided stratified log-rank tests will be carried out to compare efficacy between paclitaxel alone vs paclitaxel plus TAK228. Kaplan-Meier curves and median time to event, and the corresponding 95% confidence intervals, will be estimated adjusting by stratification factors.

Difference in ORR and CBR will be estimated using the Cochran-Mantel Haenszel chi-square test, together with the point estimates of the rates with the corresponding exact 2-sided 95% CI.

Comparison between treatment groups of CA125 responses according to Gynaecologic Cancer Group (GCIG) criteria will be made using a chi-square test.

At follow-up timepoints, vital signs (height, weight, blood pressure, body temperature and pulse), ECG, and laboratory parameter data will be summarised as percentage change from baseline, by cycle and by group, using descriptive statistics. Separate graphs for vital signs and ECG showing percentage change from baseline for each cycle will be used to illustrate changes by paclitaxel alone vs paclitaxel plus TAK228.

### Quality of life

The score for both quality-of-life questionnaires (QLQ-C30 and QLQ-OV28) will be calculated using the European Organisation for Research and Treatment of Cancer (EORTC) scoring system (see Additional file 3: Appendix 3).

All quality of life dimension scores will be summarised at each timepoint (of each cycle) using descriptive statistics. Mixed effect models of each dimension (15 for the QLC-30 and 7 for the QLQ-OV28, see Additional file 3: Appendix 3) of the quality of life (QoL) overall score will be used to investigate changes over time. Two models will be tested, one with a random-intercept term at the patient level (model 1), and one with both a random-intercept term at the patient level and a random slope term at cycle (visit) level (model 2). For model 1, we will use as a covariance structure one unique variance parameter per random effect, with all covariances at zero. For model 2, we will use an unstructured covariance. We will test the significance of the random slope term using the likelihood ratio test, to determine the appropriate model to be used [13]. The models will include the baseline measurement of each overall scores dimension, allocated treatment, centre, country, stratification factors and age as fixed effects; cycle (fixed effect in model 1 and random effect in model 2) and an interaction of cycle and allocated treatment. Graphs of the mean QoL dimension scores with 95% CI will be used to illustrate changes at each cycle.

### Compliance and treatment modification

Classifications with regards to compliance and treatment modification will be summarised by the number of compliers and the number of patients with unmodified treatment for paclitaxel alone and paclitaxel plus TAK228, at 80% and 90% thresholds. This will also be used in the sensitivity analysis described earlier.

If there is a difference in compliance, between the groups, then we will conduct an appropriate analysis using CACE methods that take into consideration non-compliance.

### Safety analysis

The information collected on adverse events (AEs) will be listed and summarised (frequency and percentage), at patient and AE level, by relationship to study treatment, severity, site and country. AEs will be coded using the Medical Dictionary for Regulatory Activities (MedDRA) terminology. Additionally, the most common AEs graded 3 or higher (Additional file 4: Supplement 1—Table 6) using Common Terminology Criteria for Adverse Events (CTCAE) 4.03 [14] will be summarised (by frequency and percentage) for paclitaxel alone vs paclitaxel plus TAK228. The decision on which AEs (graded 3 or higher) are most common was taken in discussion with the chief investigator for the Statistical Analysis Plan for the Interim Analysis.

Similar to AEs, the information of serious adverse events (SAEs) will be listed and summarised (frequency and percentage), at patient and SAE level, by site, category, and paclitaxel alone vs paclitaxel plus TAK228.

### COVID-19

Summaries of patient characteristics, protocol deviations and violations, will be presented for both the population recruited before COVID-19 and the population recruited during/after COVID-19 using the randomisation date as a reference point.

### Missing data

A summary table containing the reasons for missing primary outcome data and the relationship to treatment will be presented. Missing data are expected for some patients because the trial is recruiting and collecting data during the COVID-19 pandemic [15]. There will be no data imputation for missing data for either the primary endpoint or the secondary endpoints, except for the quality of life (QoL) questionnaires. If the missing data are less than 15%, we will fit a mixed effect model on the available data. If more than 15% of the data are missing, we will use a mixed effect model both without imputing the data and with multiple imputation for all participants who have missing values in the QoL questionnaires. We will present both results.

If more than 40% of the data are missing, only the complete cases will be used, as using multiple imputation when more than 40% of the cases are missing could significantly compromise the results [16].

### Missing data in Quality-of-Life Questionnaire

We will assume that the data is missing at random (MAR). The missing values of the QoL questionnaires will be imputed using multiple imputation by a chained equations method using the mi package in Stata 17. Ten imputations will be performed using a set of imputation models constructed from the outcome variables (each individual dimension score) and all the covariates (treatment, cycle, interaction of cycle*arm, centre, country, randomisation stratification factors and age). Inference for the imputed analysis models will use Rubin’s rules.

A sensitivity analysis for testing the robustness against the departure from the MAR assumption will be performed. The sensitivity analysis will uncover the bias from assuming the worst reasonable scenario that the missing mechanism is MNAR. The sensitivity analysis will adopt a pattern mixture model under the MNAR assumption [17]. This consists in imputing the missing data under the MAR assumption and then adjusting the imputation by adding the Delta-adjustment using a tipping point and using Rubin’s rules for the inference of the imputed models.

## Discussion

In this paper, we have described the statistical analysis plan (SAP) for the DICE trial. The trial is ongoing, and recruitment should be completed by the end of 2021. The SAP is written in advance of any data analysis to minimise any potential bias in the analysis.

The study will investigate one primary outcome, PFS, and nine secondary outcomes.

Moreover, we will estimate how treatment modification, compliance and missing data may have an impact on the comparison between paclitaxel alone and paclitaxel plus TAK228.

## Supplementary Information

**Additional file 1: Appendix 1.** Outcomes definitions

**Additional file 2: Appendix 2.** Interim analysis

**Additional file 3: Appendix 3.** Quality of life questionnaires

**Additional file 4: Supplement 1:** Tables. Table 1: Schedule of Assessments for Screening. Table 2: Schedule of Assessments for: Weekly Paclitaxel alone. Table 3: Schedule of Assessments for Weekly Paclitaxel plus TAK228. Table 4: Baseline Characteristic Variables. Table 5: Laboratory parameters. Table 6: Listing of “Most Common” Adverse Events.

**Additional file 5: Supplement 2:** Figure. Figure 1: Planned CONSORT Diagram.

## Data Availability

The chief investigator is the custodian of the final trial dataset, which may be shared according to both the contractual agreement with the funder and relevant standard operating procedures.

## Abbreviations

AE: Adverse event
AKT: Protein kinase B
CA125: Cancer antigen 125
CBR: Clinical benefit rate
CI: Chief investigator
CONSORT: Consolidate Standards of Reporting Trial
CTCAE: Common Terminology Criteria for Adverse Events
DICE: Dual mTorc Inhibition in advanced/recurrent epithelial ovarian, fallopian tube or primary peritoneal cancer (of clear cell, endometrioid and high grade serous type, and carcinosarcoma) trial
EORTC: European Organisation for Research and Treatment of Cancer
eCRF: Electronic case report form
GCP: Good Clinical Practice
IDMC: Independent Data Monitoring Committee
ITT: Intention to treat
MAR: Missing at random
MedDRA: Medical Dictionary for Regulatory Activities
MRI: Magnetic resonance imaging
mTOR: Mammalian target of rapamycin
OF: O’Brian Fleming method
ORR: Overall response rate
OS: Overall survival
PFS: Progression-free survival
PI3K: Phosphoinositide 3-kinase
QLQ-C30: Quality of life core questionnaire
QLQ-OV28: Supplementary quality of life questionnaire for ovarian cancer
RECIST: Response Evaluation Criteria in Solid Tumours
SAE: Serious adverse event
SAP: Statistical analysis plan
SD: Standard deviation
TORC1/2: Target of rapamycin complex 1/2
TTP: Time to progression
