# Supplementary material for: Statistical analysis plan for the Dual mTorc Inhibition in advanCed/recurrent Epithelial ovarian, fallopian tube or primary peritoneal cancer (of clear cell, endometrioid and high-grade serous type, and carcinosarcoma) trial (DICE)
Source: Trials. 2022 Jan 5;23:13. doi: 10.1186/s13063-021-05669-9 (PMC8728702; doi:10.1186/s13063-021-05669-9)
Supplement: Supplementary file 1 — Additional file 1: Appendix 1. Outcomes definitions [file 13063_2021_5669_MOESM1_ESM.docx]

# **Appendix 1: Outcomes definitions**

**Table 1 Outcomes definitions**

| Primary outcome | **Outcome** | **Definition** | **Censoring** |
| --- | --- | --- | --- |
|  | Progression Free Survival  (PFS) | Time in months from randomisation to first evidence of disease progression or death due to any cause, whichever occurs first, as assessed by RECIST v1.1. Patients will be followed-up until the completion of the study. | Patients who do not have baseline disease assessment are left censored, whereas patients are right censored if:   - The patient is alive and does not have documentation of disease progression before the date of completion of the study - The patient withdraws from the trial during the study treatment period and asks to stop being followed up - A patient is lost to follow-up |
| Secondary outcomes -Time to event | Progression Free Survival at 24 weeks (PFS at 24 weeks) | Time in months from randomisation to first evidence of disease progression or death due to any cause at 24 weeks, whichever occurs first, as assessed by RECIST v1.1.  Patients will be followed-up until the completion of the study. | Patients who do not have baseline disease assessment are left censored, whereas patients are right censored if:   - The patient is alive and does not have documentation of disease progression at 24 weeks - The patient withdraws from the trial during the study treatment period within 24 weeks and asks to stop being followed up - A patient is lost to follow-up at 24 weeks |
|  | Duration of response (DoR) | Time from study entry to change in response from CR or PR or stable disease (SD) to progressive disease (PD) as assessed by RECIST v1.1. | Patients who do not have baseline disease assessment are left censored, whereas patients are right censored if:   - The patient is alive and does not have documentation of disease progression before the date of completion of the study. - Withdrawal or loss to follow up before change in response. - Death due to any cause other than disease progression. |
|  | Time to progression (TTP) | Time from study entry to first evidence of disease progression or death due to any cause, whichever occurs first, as assessed by RECIST v1.1. Patients will be followed-up until completion of study. | Patients who do not have baseline disease assessment are left censored, whereas patients are right censored if:   - The patient is alive and does not have documentation of disease progression before the date of completion of the study. - The patient withdraws from the trial during the study treatment period and asks to stop being followed up. - A patient is lost to follow-up. |
|  | Overall Survival (OS) | Overall survival (OS) defined as time from study entry to death due to any cause or study termination, whichever occurs first. Patients will be followed-up until completion of study | Patients who do not have baseline disease assessment are left censored, whereas patients are right censored if:   - The patient is alive and does not have documentation of disease progression before the date of completion of the study and asks to stop being followed up. - Withdrawal or loss to follow up before death. - A patient is lost to follow-up. |
| **Other secondary outcomes definitions** | | | |
| Overall response rate (ORR) | | ORR is defined as Complete (CR) or Partial Response (PR) (as assessed by RECIST v1.1). The Best Overall Response will be used when Confirmation is Required. | |
| Clinical Benefit Rate (CBR) | | CBR (as assessed by RECIST v1.1) is defined as Complete Response (CR), Partial Response (PR) or Stable Disease (SD) at > 4 months. | |
| CA125 response according to GCIG criteria | | A response according to GCIG criteria has occurred if there is at least a 50% reduction in CA125 levels from a pre-treatment sample. The response must be confirmed and maintained for at least 28 days. Patients can be evaluated according to CA125 only if they have a pre-treatment sample that is at least twice the upper limit of normal and within 2 weeks prior to starting treatment. | |
