# Supplementary material for: Statistical analysis plan for the Dual mTorc Inhibition in advanCed/recurrent Epithelial ovarian, fallopian tube or primary peritoneal cancer (of clear cell, endometrioid and high-grade serous type, and carcinosarcoma) trial (DICE)
Source: Trials. 2022 Jan 5;23:13. doi: 10.1186/s13063-021-05669-9 (PMC8728702; doi:10.1186/s13063-021-05669-9)
Supplement: Supplementary file 2 — Additional file 2: Appendix 2. Interim analysis [file 13063_2021_5669_MOESM2_ESM.docx]

# **Appendix 2: Interim analysis**

## Objective

The purpose of the interim analysis was to allow early stopping of the study for futility and safety (toxicity). The formal stopping rules for futility were defined using the O’Brien-Fleming (OF) method (1) while the safety rules were defined using the Pocock method (2) (3).

## Primary endpoint for the interim analysis

The primary endpoint was progression free survival (PFS) as assessed by RECIST v1.1 (4), defined as time from study entry (randomisation) to first evidence of disease progression or death due to any cause, whichever occurred first.

### Definition of disease progression

Disease progression is a worsening of a patient’s condition attributable to the disease for which the study medication is being given. This may be an increase in severity of the disease or increase in the symptoms of the disease. The development of new, or progression of existing metastasis to the primary cancer under study, should also be considered disease progression.

## Safety (toxicity) endpoint

The safety (toxicity) was evaluated by comparing the proportion of patients, for paclitaxel alone and paclitaxel plus TAK228, who experience one of the most common AEs graded 3 or higher. AE classification was assessed using the National Cancer Institute CTCAE version 4.03 (5) (June 2010) grading scale (0 to 5) for all AEs with an assigned CTCAE term. The list of most common AEs was discussed and agreed with the CI. This list can be found in Supplement 1 – Table 6.

## Stratification factors

Treatment assignment is stratified based on histology: non-serous (clear cell or endometrioid) vs serous (high grade serous or carcinosarcoma) cancer, the number of prior lines of chemotherapy (≤ 2 vs > 2 lines) and prior taxane interval (< 6 months vs ≥ 6 months or no prior taxane).

## Futility stopping rules

The objective of the futility analysis based on the primary end point (PFS) is to stop enrolment to the study if there is sufficient evidence that the treatment arm (Paclitaxel+TAK228) is not improving outcomes compared to the control arm (Paclitaxel alone).

The O’Brien Fleming sequential method (2) was selected as it allows the significance level of the final analysis to be near the desired overall significance alpha (Type I error) of 10% and beta (Type II error) of 20%.

The futility O’Brian-Fleming boundaries (one-sided hypothesis test) were obtained using the SEQDESIGN sample size calculation procedure in SAS. The information used for this (see Table A1) was:

- Significance level of 10% (alpha)
- Type II error of 20% (beta)
- Median survival time for both the paclitaxel and TAK228 together (𝝀_𝒕_) = 6.15months
- Median survival time for the paclitaxel alone (𝝀_𝒄_) = 4 months
- Accrual time = 18 months
- Follow-up time = 6 months.

Table A1: One-sided (Lower) Futility Stopping Boundary of the O’Brien-Fleming method (OF) for PFS for rejecting the Alternative Hypothesis (H_a_).

| Analysis Number | Number of Events (% Information) | Standardized boundary^1^ | Nominal Significance level (boundary value) |
| --- | --- | --- | --- |
| 1 (interim) | 49 (planned) | -0.20706 | 0.41798 |
| 2 (final) | 102 | -1.22626 | 0.11005 |

^1^ O’Brien-Fleming boundaries to reject Ha.

The futility analysis was conducted using the significance level (boundary value) of 0.41798 for the interim (49 PFS cases) and with a significance level (boundary value) of 0.11005 for the final analysis (102 PFS cases). Stopping the trial early for futility is considered if the p-value for PFS is less than the alpha boundary (rejecting the alternative hypothesis) at the interim analysis.

## Sample sizes adjustment due to interim analysis

As DICE trial was allowed to stop early for futility, there was a risk of losing power (3) (6). The O’Brien-Fleming (OF) method which is part of the group sequential methods address this problem directly by maintaining both the power and type I error at the desired rates. The cost of this approach is an increase in the maximum sample size.

The output of SEQDESIGN which provides the stopping boundaries also generates an inflation factor (“Max Information”) which is how much the fixed number of progression events needed to inflate by.

Max Information (Percent of Fixed Sample) = 1.054887 *97 **≈** 102

Given the new total of 102 events and the original total of 97 events, 5 events more than the original are needed. To attain 102 events, the adjusted sample size is 124 patients (62 by treatment arm). This adjusted sample size was obtained using the INPUTNOBS option of SEQDESIGN.

## Safety (toxicity) stopping rules

The objective of the safety stopping rules was to stop if the most common adverse events (AEs) (see Supplement 1 – Table 6) graded as 3 or higher were more frequent in Paclitaxel + TAK228 than in the Paclitaxel alone.

The threshold for the interim analysis is based on the Pocock sequential approach (2) to maintain the overall Type I error rate or alpha of 5%. This method was selected as it divides alpha equally amongst the two analyses planned for the DICE trial.

The safety (Pocock) boundaries in Table A2 below were obtained using the SEQDESIGN procedure in SAS, using a significance level (alpha) of 5%, and Type II error (beta) of 10% for a one-sided test.

Table A2 One-sided (Upper) Safety (toxicity) Stopping Boundaries (Pocock) for the chi-square test for Rejecting the Null hypothesis (H_0_)

| Analysis Number | Number of Events (% Information) | Standardized boundary^1^ | ^2^Nominal Significance level (alpha (Type I error)) |
| --- | --- | --- | --- |
| 1(Interim) | 49 | 1.87542 | 0.03037 |
| 2(Final) | 102 | 1.87542 | 0.03037 |

^1^ Pocock method to reject Ho

The safety (toxicity) analysis was conducted using the significance level (alpha) of 0.03037 for the interim (49 PFS cases) and with a significance level (alpha) of 0.03037 for the final analysis (102 PFS cases). Stopping the trial early for safety (toxicity) is considered if the p-value of the Chi-square test comparing the proportions of the most common AEs (see Supplement 1 – Table 6) graded as 3 or higher between paclitaxel alone vs paclitaxel plus TAK228 is less than the significance level (alpha) boundary (rejecting the null hypothesis) at the interim analysis.

# Statistical methodology

## Primary endpoint analysis

We used the Kaplan Meier method to estimate the overall PFS.

PFS was censored for patients who:

- Had no baseline disease assessment (right censored)
- Died from any cause or experienced disease progression after more than one missed disease assessment visit (left censored)
- Were alive and did not have documentation of disease progression before the data analysis cut-off date (left censored)

PFS was displayed using the Kaplan-Meier curves for each treatment arm. Median PFS was estimated for each treatment group and overall. The primary inferential comparison between treatment groups used a log-rank test adjusting for the randomisation stratification factors. The number and percentage of subjects who progressed, or were censored (for the reasons above), were reported.

## Safety (toxicity) endpoint analysis

The safety analysis estimated the difference between paclitaxel alone vs paclitaxel plus TAK228 in the proportions of most common AEs (see Supplement 1 – Table 6), graded 3 or higher, using a Chi-square test.

# Interim analysis results

In the interim analysis, the p-value generated by the log-rank test comparing the primary outcome (PFS) between paclitaxel alone vs paclitaxel plus TAK228 was compared to the significance level (boundary value) of 0.41798 for futility.

The p-value generated by the Chi-square test, assessing the proportions of most common AEs (see Supplement 1 – Table 6) classified as grade 3 or higher between paclitaxel alone vs paclitaxel plus TAK228, was compared to the alpha stopping boundary of 0.03037 for safety.

The Independent Data Monitoring Committee (IDMC) assessed that there was no reason to prematurely stop the trial for concerns over futility or safety. The IDMC advised to continue the study to completion.

# References

1. *A Multiple Testing Procedure for Clinical Trials.* **Fleming, Peter C. O'Brien and Thomas R.** 3, Sep 1979, International Biometric Society, Vol. 35, pp. 549-556.

2. **Jennison, Christopher and Turnbull, Bruce W.** *Group Sequential Methods with Applications to Clinical Trials.* s.l. : Chapman and Hall/CRC, 2000. 9780849303166.

3. *Assessment of futility in clinical trials.* **Snapinn, Steven, et al.** 4, Oct-Dec 2006, Pharmaceutical Statistics, Vol. 5, pp. 273-81.

4. *New response evaluation criteria in solid tumours: Revised RECIST guideline (version 1.1).* **Eisenhauer, E. A., et al.** 2009, European Journal of Cancer, Vol. 45, pp. 228-247.

5. **U.S.DEPARTMENT OF HEALTH AND HUMAN SERVICES.** Common Terminology Criteria for Adverse Events (CTCAE) v4.03. [Online] 14 June 2010. https://evs.nci.nih.gov/ftp1/CTCAE/CTCAE_4.03/CTCAE_4.03_2010-06-14_QuickReference_5x7.pdf.

6. *Choice of futility boundaries for group sequential designs with two endpoints.* **Schüler, Svenja, Kieser, Meinhard and Rauch, Geraldine.** 1, 2017, BMC Medical Research Methodology, Vol. 17.
