# Supplementary material for: Statistical analysis plan for the Dual mTorc Inhibition in advanCed/recurrent Epithelial ovarian, fallopian tube or primary peritoneal cancer (of clear cell, endometrioid and high-grade serous type, and carcinosarcoma) trial (DICE)
Source: Trials. 2022 Jan 5;23:13. doi: 10.1186/s13063-021-05669-9 (PMC8728702; doi:10.1186/s13063-021-05669-9)
Supplement: Supplementary file 3 — Additional file 3: Appendix 3. Quality of life questionnaires [file 13063_2021_5669_MOESM3_ESM.docx]

# **Appendix 3: Quality of life questionnaires**

## EORTC QLQ-C30 core questionnaire

The EORTC QLA-C30 version 3.0 questionnaire (1) has been developed to assess the quality of life of cancer patients. This a copyrighted instrument, which has been translated and validated.

The QLQ-C30 is composed of both multi-item scales and single-item measures. It consists of 30 items assessing five functional scales, three symptom scales, a global health status / QoL scale, and six single items. All the scales and single-item measures range in score from 0 to 100. A higher scale score represents a higher response level.

Table B1: Scoring the QLQ-C30 version 3.0

| **EORTC QLQ-C30 Scores**  **dimensions** | **Abbreviation** | **Number of questions** | **Question range*** | **Version 3.0 question number (q)** |
| --- | --- | --- | --- | --- |
| ***Global Health status / QoL*** |  |  |  |  |
| Global health status/QoL (revised) † | QL2 | 2 | 6 | 29,30 |
| ***Functional scales*** |  |  |  |  |
| Physical Functioning (revised) † | PF2 | 5 | 3 | 1 to 5 |
| Role Functioning (revised) † | RF2 | 2 | 3 | 6,7 |
| Emotional Functioning | EF | 4 | 3 | 21 to 24 |
| Cognitive Functioning | CF | 2 | 3 | 20,25 |
| Social Functioning | SF | 2 | 3 | 26,27 |
| ***Symptom scales/items*** |  |  |  |  |
| Fatigue | FA | 3 | 3 | 10,12,18 |
| Nausea and Vomiting | NV | 2 | 3 | 14,15 |
| Pain | PA | 2 | 3 | 9,19 |
| Dyspnoea | DY | 1 | 3 | 8 |
| Insomnia | SL | 1 | 3 | 11 |
| Appetite loss | AP | 1 | 3 | 13 |
| Constipation | CO | 1 | 3 | 16 |
| Diarrhoea | DI | 1 | 3 | 17 |
| Financial difficulties | FI | 1 | 3 | 28 |

* Question range is the difference between the possible maximum and the minimum response to individual questions; most questions take values from 1 to 4, giving range = 3.

† (revised) dimensions are those that have been changed since version 1.0, and their short names are indicated in this manual by a suffix “2” – for example, PF2.

## Scoring process

Every dimension (column 1) in Table B1 has a specific number of questions assigned that are used to obtain the score (“Version 3.0 question number”).

For instance, for Global health status (QL2) dimension, there are two questions (number 29 and 30) used to obtain the score. The raw score for each dimension is calculated by averaging all the questions assigned to the specific dimension, as illustrated in Table B2.

For example, for QL2 we obtain the raw score by adding q29 and q30 and dividing by two.

Raw score (RS)= (q29+q30)/2

Once the raw score is obtained a linear transformation is applied. In the case of Global Health status (QL2) the transformation (Table B2) is:

*S={(RS-1)/range}x100*

The scoring process for the dimensions (Table B1) is the same in all cases. A summary of the process is described in Table B2 below.

Table B2: Technical summary for scoring QLQ-C30 questionnaire

| **Technical Summary** |  |
| --- | --- |
|  |  |
| In practical terms, if question Items *q_1_,q*_2_*,. . . q_n_* are included in a scale, the procedure is as follows: | |
| **Raw score** |  |
| Calculate the raw score | *Raw Score (RS)=(q_1_+q_2_+…+q_n_)/n* |
|  |  |
| **Linear Transformation** |  |
| Apply the linear transformation to 0-100 to obtain the score S, | |
| Functional scales: | *S={1-(RS-1)/range}x100* |
| Symptom scales/items: | *S={(RS-1)/range}x100* |
| Global Heath Status/QoL: | *S={(RS-1)/range}x100* |
|  |  |
| ***Range*** is the difference between the maximum possible value of ***RS*** and the minimum possible value | |

## EORTC QLQ-OV28 ovarian cancer

The QLQ-OV28 is a supplementary questionnaire (which is usually employed in conjunction with the QLQ-C30. The EORTC QLQ-OV28 assesses the quality of life of women with ovarian cancer in clinical trials. It was designed for patients with local or advanced disease who receive treatment by surgery with or without chemotherapy. It consists of 28 items assessing abdominal/GI symptoms, peripheral neuropathy, other chemotherapy side effects, hormonal symptoms, body image, attitudes to disease/treatment, and sexuality.

## Scoring of the ovarian cancer module

The scoring approach for the ovarian cancer module is identical in principle to that used for the scales/items of the EORTC QLQ-C30 (see Table B2). The scale structure proposed in Table B3 is based on preliminary scaling analysis of the first 24 items of the module, i.e., excluding items 25-28 on sexuality (1).

Table B3: Scoring the QLQ-OV28

| **EORTC QLQ-OV28 Scores**  **dimensions** | **Number of questions** | **Item range*** | **Version 3.0 question number (q)** |
| --- | --- | --- | --- |
| ***Symptom scales / items*** |  |  |  |
| Abdominal/gastrointestinal symptoms | 6 | 3 | 1-6 |
| Peripheral neuropathy | 2 | 3 | 11,12 |
| Hormonal/menopausal | 2 | 3 | 18,19 |
| Body imagen | 2 | 3 | 20,21 |
| Attitude to disease and treatment | 3 | 3 | 22,23,24 |
| chemotherapy side-effects | 5 | 3 | 13-17 |
| Other single items | 4 | 3 | 07-10 |
| Sexuality |  |  | 25-28 |

# References

1. ***EORTC Quality of Life.* [Online] https://qol.eortc.org/.**
