# Supplementary material for: Statistical analysis plan for the Dual mTorc Inhibition in advanCed/recurrent Epithelial ovarian, fallopian tube or primary peritoneal cancer (of clear cell, endometrioid and high-grade serous type, and carcinosarcoma) trial (DICE)
Source: Trials. 2022 Jan 5;23:13. doi: 10.1186/s13063-021-05669-9 (PMC8728702; doi:10.1186/s13063-021-05669-9)
Supplement: Supplementary file 4 — Additional file 4: Supplement 1: Tables. Table 1: Schedule of Assessments for Screening. Table 2: Schedule of Assessments for: Weekly Paclitaxel alone. Table 3: Schedule of Assessments for Weekly Paclitaxel plus TAK228. Table 4: Baseline Characteristic Variables. Table 5: Laboratory parameters. Table 6: Listing of “Most Common” Adverse Events. [file 13063_2021_5669_MOESM4_ESM.docx]

# Supplement 1: Tables

Table 1: Schedule of Assessments for Screening

| Time-point→ | Screening/Baseline | |
| --- | --- | --- |
| Assessments↓ | Within 28 days | Within 14 days |
| Informed Consent | X |  |
| Inclusion/exclusion criteria | X |  |
| Demographics | X |  |
| Medical History | X |  |
| German sites only: screening for HIV and hepatitis | X |  |
| Vital signs |  | X |
| Eastern Cooperative Oncology Group (ECOG) Performance Status |  | X |
| Complete Physical Examination |  | X |
| 12-lead electrocardiogram (ECG) | X |  |
| Haematology |  | X |
| Biochemistry |  | X |
| Coagulation |  | X |
| Fasting Serum Glucose |  | X |
| Glycosylated Haemoglobin (HbA1c) |  | X |
| Fasting Lipid Profile |  | X |
| Urinalysis |  | X |
| Creatinine clearance based on CockcroftGault estimate, Wright Formula or urine collection |  | X |
| Blood Serum Pregnancy Test |  | X |
| Research Blood Sample for genomic DNA |  | X |
| Archival Tumour Tissue | X |  |
| Radiological imaging assessment (CT with contrast/MRI chest, abdomen and pelvis) | X |  |
| Cancer antigen 125 (CA125) | X |  |
| Research Fresh Tumour Biopsy, if judged technically feasible by radiologist, unless the local site is unable to collect the sample due to COVID-19 capacity restrictions |  | X |
| Quality of Life Questionnaires:  Quality of life core questionnaire (EORTC QLQC30) and supplementary quality of life questionnaire for ovarian cancer (EORTC QLQ-OV28) | X |  |
| Adverse Events (NCI CTCAE version 4.03) |  | X |
| Concomitant Medications |  | X |
| Randomisation |  | X |

Table 2: Schedule of Assessments for: Weekly Paclitaxel alone

| Time-point→ | Each Treatment Cycle | | | End of Treatment Visit | Follow-up |
| --- | --- | --- | --- | --- | --- |
| Assessments↓ | Day 1 | Day 8 | Day 15 | 30 days post last treatment (±5 days) | Every 3 months (±5 days) |
| Vital signs | X | X | X | X |  |
| Eastern Cooperative Oncology Group (ECOG) Performance Status | X | X | X | X |  |
| Physical Examination | X | X | X | X |  |
| Haematology | X | X | X | X |  |
| Biochemistry | X | X | X | X |  |
| Urinalysis | X |  | X  Cycles 1 and 2 only | X |  |
| Pregnancy Test | X |  |  | X |  |
| Research Blood Sample for ctDNA | X |  |  | X |  |
| Radiological imaging assessment (CT with contrast/MRI chest, abdomen and pelvis) | Every 2 cycles/8 weeks (± 7 days) | | | X | X |
| Cancer antigen 125 (CA125) | X^1^ |  |  | X | X |
| Paclitaxel Administration | X | X | X |  |  |
| Quality of Life Questionnaires (EORTC QLQ-C30 and EORTC QLQ-OV28) | X |  |  | X |  |
| Adverse Events (NCI CTCAE version 4.03) | X | X | X | X | X |
| Concomitant Medications | X | X | X | X |  |
| Record of Further Treatment |  |  |  |  | X |
| Record of Overall Survival |  |  |  |  | X |

Table 3: Schedule of Assessments for Weekly Paclitaxel plus TAK228

| Time-point→ | Each Treatment Cycle | | | | End of Treatment Visit | Follow-up |
| --- | --- | --- | --- | --- | --- | --- |
| Assessments↓ | Day 1 | Day 2 (Cycles 1 & 2 only) | Day 8 | Day 15 | 30 days post last treatment (±5 days) | Every 3 months (±5 days) |
| Vital signs | X^2^ |  | X | X | X |  |
| Eastern Cooperative Oncology Group (ECOG) Performance Status | X^2^ |  | X | X | X |  |
| Physical Examination | X^2^ |  | X | X | X |  |
| 12-lead ECG | X |  |  |  | X |  |
| Haematology | X |  | X | X | X |  |
| Biochemistry | X |  | X | X | X |  |
| Coagulation | X^2^ |  |  |  | X |  |
| Fasting Serum Glucose | X |  |  |  | X |  |
| Glycosylated Haemoglobin (HbA1c) | X^2^ |  |  |  |  |  |
| Fasting Lipid Profile | X^2^ |  |  |  | X |  |
| Urinalysis | X^2^ |  |  | X  Cycles 1 and 2 only | X |  |
| Pregnancy Test | X^2^ |  |  |  | X |  |
| Research Blood Sample for Circulating tumour DNA (ctDNA) | X |  |  |  | X |  |
| Radiological imaging assessment (CT with contrast/MRI chest, abdomen and pelvis) | Every 2 cycles/8 weeks (± 7 days) | | | | X^15^ | X^16^ |
| Cancer antigen 125 (CA125) | X^2^ |  |  |  | X | X |
| In-Home Daily Fasting Glucose Monitoring | X | X | X | X |  |  |
| Paclitaxel Administration | X | X | X | X |  |  |
| TAK228 Administration/Compliance: administration at days 2-4, 9-11, 16-18 and 23-25 per cycle | X | X | X | X |  |  |
| Quality of Life Questionnaires (EORTC QLQ-C30 and EORTC QLQ-OV28) | X |  |  |  | X |  |
| Adverse Events (NCI CTCAE version 4.03) | X | X | X | X | X | X |
| Concomitant Medications | X | X | X | X | X |  |
| Record of Further Treatment |  |  |  |  |  | X |
| Record of Overall Survival |  |  |  |  |  | X |

Table 4: Baseline Characteristic Variables

| Age |
| --- |
| Ethnicity |
| Eastern Cooperative Oncology Group (ECOG) Performance Status |
| Primary Site |
| Histology of Tumour |
| FIGO Cancer Stage |
| Tumour Grade |
| Treatment history:   - Surgical History - Radiotherapy History - Chemotherapy History - Endocrine Therapy History - Target Therapy History |

Table 5: Laboratory parameters

| Haematology: White blood cell count (WBC) with differential (to include neutrophils, lymphocytes), haemoglobin (Hgb), absolute neutrophil count (ANC) and platelet count, Glycosylated haemoglobin (HbA1c) |
| --- |
| Coagulation: International normalized ratio (INR) and activated partial thromboplastin time (aPTT) |
| Biochemistry: Chem 7 (sodium [Na], potassium [K], chloride [Cl], bicarbonate [CO2], urea, creatinine [Cr], glucose), liver function tests (LFTs - ALT, AST, alkaline phosphatase, total bilirubin, direct bilirubin), lactate dehydrogenase (LDH), total protein, calcium, phosphate, and magnesium |
| Fasting lipid profile: Total cholesterol, high density lipoprotein cholesterol (HDL-C), low density lipoprotein cholesterol (LDL-C), and triglycerides, fasting serum glucose; |
| Cancer antigen 125 (CA125) |

Table 6: Listing of “Most Common” Adverse Events

| Fatigue/Lethargy |
| --- |
| Nausea |
| Diarrhoea |
| Anaemia |
| Abdominal Pain |
| Alopecia |
| Constipation |
| Vomiting |
| Anorexia |
| Neutropenia |
| Hypomagnesemia |
| Dyspnoea |
| Peripheral Neuropathy |
| Hypertension |
| Fever/Pyrexia |
| Cough/Dry cough |
| Shortness of breath |
| White blood cell decreases |
| Hyperglycaemia |
| Ascites |
| Urinary Tract Infection |
| Bloating |
| Headache |
| Mouth ulcer/Mucositis |
| Back pain |
| Dry mouth |
| Neurotoxicity |
