# Supplementary figures and images for: Statistical analysis plan for the Dual mTorc Inhibition in advanCed/recurrent Epithelial ovarian, fallopian tube or primary peritoneal cancer (of clear cell, endometrioid and high-grade serous type, and carcinosarcoma) trial (DICE)
Source: Trials. 2022 Jan 5;23:13. doi: 10.1186/s13063-021-05669-9 (PMC8728702; doi:10.1186/s13063-021-05669-9)

Supplement 2

Figure 1: Planned CONSORT Diagram
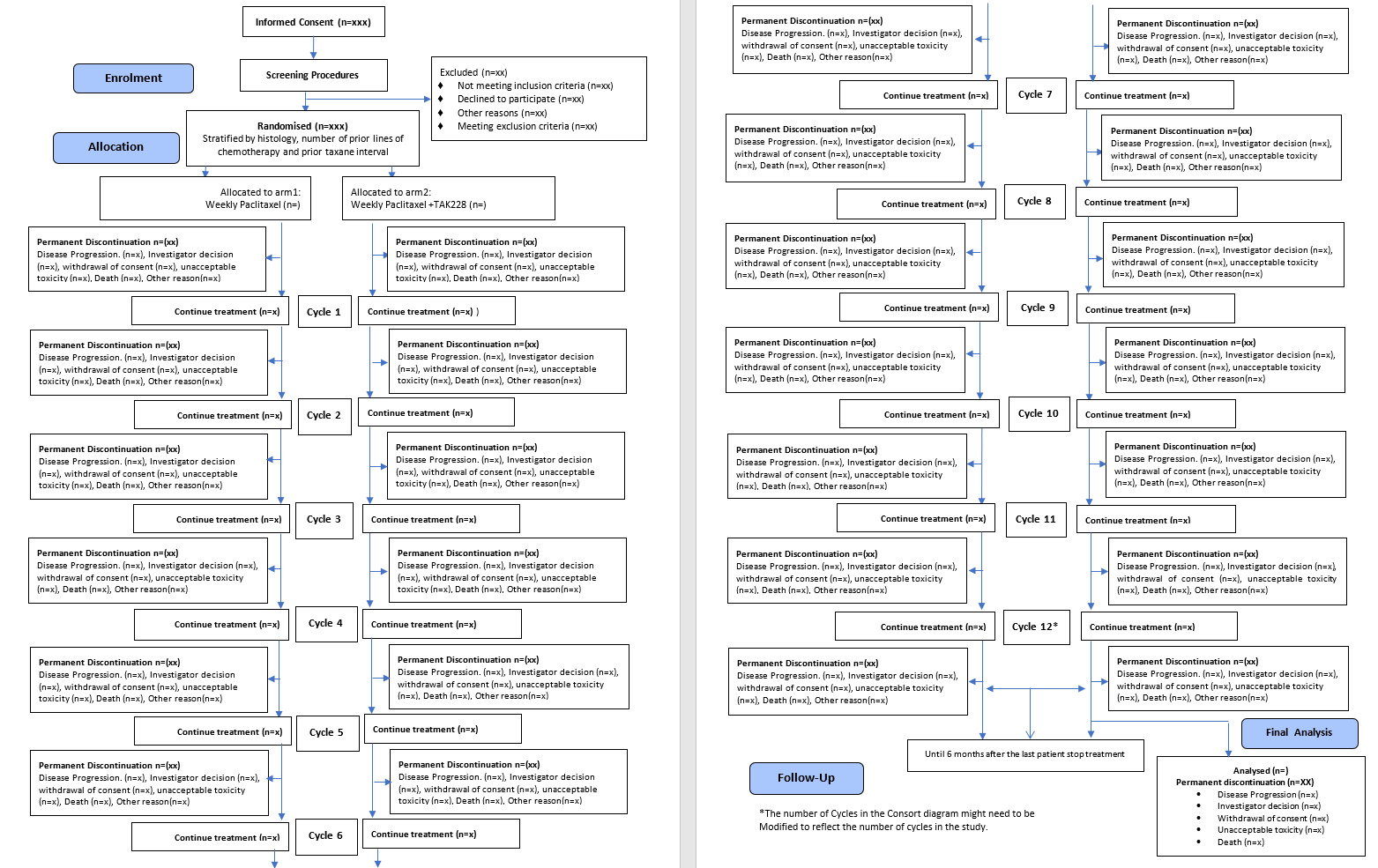

Supplement: Supplementary file 5 — Additional file 5: Supplement 2: Figure. Figure 1: Planned CONSORT Diagram. [file 13063_2021_5669_MOESM5_ESM.docx]
